# Nivolumab plus regorafenib in patients with small bowel adenocarcinoma: A case report

**DOI:** 10.1097/MD.0000000000024295

**Published:** 2021-01-29

**Authors:** Gairong Zhang, Li Lin, Dapeng Dong, Hui Qiu, Tao Liu, Li Lian, Ge Shen

**Affiliations:** a Department of Oncology, Beijing Huian Hospital of Integrated TCM and Western Medicine; b Department of Oncology, Peking University International Hospital, the 8th Clinical Medical College; c Department of Oncology, Beijing Fengtai You’anmen Hospital, Beijing, China.

**Keywords:** case report, duodenum adenocarcinoma, immune checkpoint inhibitors, nivolumab, regorafenib, small bowel adenocarcinoma

## Abstract

**Introduction:**

Small bowel adenocarcinomas (SBAs) are rare cancers that have a distinct clinical characteristic and genetic profile. The only potentially curative treatment for localized SBAs is surgery, and treatment options are limited for patients in the advanced stage of disease.

**Patient concerns:**

A 39-year-old woman presented in October 2015 with a complaint of persistent vomiting for 8 months.

**Diagnosis:**

The patient had obstruction caused by a 3 × 2 cm mass at the ascending part of the duodenum and suspected metastasis in the right adnexal region. Postoperative pathology showed a moderately differentiated adenocarcinoma with serosal invasion. The diagnosis was stage IV duodenum adenocarcinoma with right adnexal metastasis.

**Interventions:**

After the failure of multi-line treatment with chemotherapy and targeted therapy, she was treated with the immune checkpoint inhibitor nivolumab plus regorafenib.

**Outcomes:**

Disease control lasted for 15 months with markedly improved symptoms.

**Conclusion:**

To the best of our knowledge, this is the first case of small bowel adenocarcinoma that has been treated with nivolumab combined with regorafenib. This case highlights the potential efficacy of combining nivolumab and regorafenib in the treatment of SBAs.

## 1 Introduction

Small bowel adenocarcinomas (SBAs) are a rare type of cancer that accounts for only 3% of all gastrointestinal malignancies, about 1/100 to 1/50 the rate of colon cancer.^[1,2]^ Most SBAs are located in the duodenum (57%–60.6%), followed by the jejunum (20.7%–29%) and ileum (10%–18.7%).^[3,4]^ Endoscopic diagnosis is difficult due to the anatomical features. Non-specific symptoms can lead to delayed diagnosis so that most SBA patients are not diagnosed until the advanced stage.^[5]^

Advanced SBAs have a poor prognosis with a median overall survival (OS) of around 11.1 to 13.8 months.^[4,6,7]^ In addition, duodenum tumor is associated with survival rate lower to that of jejunum/ileum tumor.^[8]^ Surgery is most likely to be the best curative treatment for SBAs. As for duodenal adenocarcinoma, the 5-year OS rate was 46% after curative resection, compared with 1% in palliative-treated patients.^[9]^ For systemic therapy, several chemotherapy regimens, including eucovorin calcium, 5-fluorouracil, and oxaliplatin (FOLFOX), capecitabine and oxalipltin (CAPEOX), and folinic acid, 5-fluorouracil, oxaliplatin and irinotecan, have been tested and recommended, yielding objective response rates (ORRs) of around 37.5% to 50%.^[10–13]^ However, given the rarity of this tumor, evidence levels for treatment are insufficient, which are mostly based on single-arm prospective study with small sample size, retrospective analyses, case reports, and case series.

Here, we report a female patient with SBA treated with nivolumab plus regorafenib after failure of multi-line systemic treatment.

## 2 Case presentation

A 39-year-old woman presented in October 2015 with a complaint of persistent vomiting for 8 months. No significant past medical history or family history (including cancer) was noted. The result of the patient's physical examination was normal and indexes for laboratory testing were within the normal range, including tumor markers, such as carbohydrate antigen 19–9 (CA19–9) and carcinoembryonic antigen (CEA). Endoscopy revealed annulus protuberance near the horizontal portion of the duodenum, where abnormally increased metabolic activity was observed by positron emission computed tomography (PET-CT). PET-CT also demonstrated suspected metastasis in the right adnexal region. Subsequent surgical resection found obstruction caused by a 3x2 cm mass in the ascending section of the duodenum. Postoperative pathology showed a well-moderately differentiated adenocarcinoma with serosal invasion. CK7(–), CK20 (+), and CDX-2 (+) were identified by immunochemistry. Next-generation sequencing indicated a microsatellite stable disease with mutation of BRAF (N581S), KRAS, and PIK3CA (E545K). The diagnosis was thus a stage IV duodenum adenocarcinoma with right adnexal metastasis.

**Figure 1:**
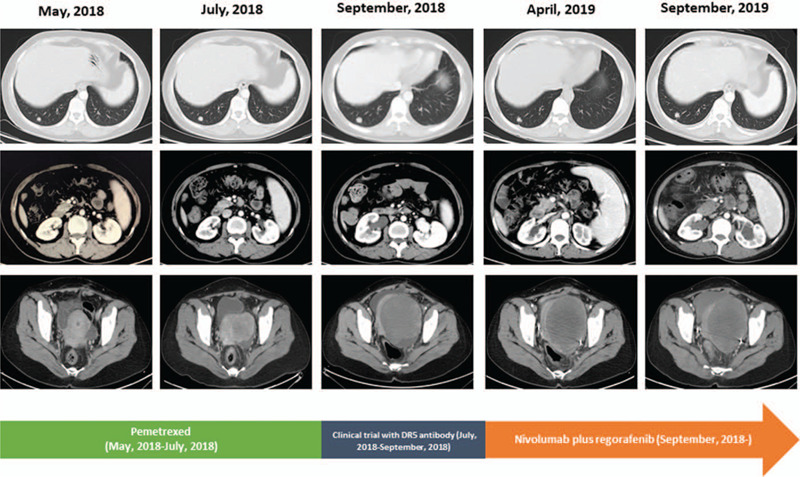
computed tomography images showing the changes of masses in lung, abdomen, and pelvis during treatment course.

**Figure 2:**
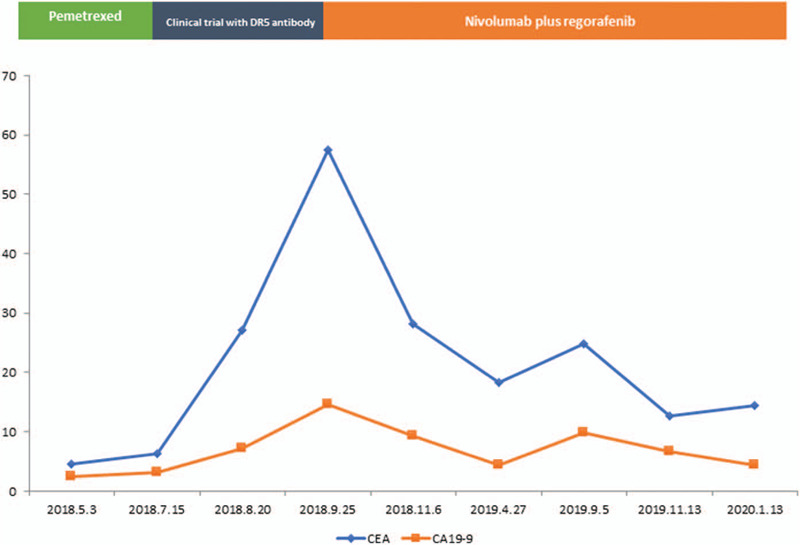
The transition of the treatment course and the tumor marker in time series. CEA (blue) = carcinoembryonic antigen, CA19–9 (red) = carbohydrate antigen 19–9.

She was then administered with capecitabine and oxaliplatin regimen every 3 weeks. After 9 courses, PET-CT (June 2016) showed metastasis with nodules in the right lower abdomen and enlarged lesion in the right adnexal region. We switched to second-line treatment with S-1 plus irinotecan every 2 weeks, and observed progression after 6 courses (September 2016). In November 2016, the patient underwent bilateral ovariectomy and peritoneal cytoreduction due to abdominal pain and bloating, followed by consolidation chemotherapy with FOLFOX regimen between December 2016 and August 2017. Computed tomography (CT) and PET-CT scans displayed bilateral lung metastases in September 2017. Oral regorafenib 160 mg was given once daily, followed by dose reduction to 120 mg due to grade 3 hand-foot syndrome and diarrhea. However, she still suffered from grade 3 hand-foot syndrome and grade 1 diarrhea at the reduced dose. With stable disease as the response, she progressed on regorafenib in March 2018 with worsening lung metastases and new metastases in the abdomen and pelvis. As shown in Figures 1 and 2, rapid progression was seen with enlarged lesions, multiple metastases, and increased CEA and CA19–9 levels after treatment failure on pemetrexed monotherapy and DR5 antibody (clinical trial).

Re-biopsy using a 288-gene panel revealed mutations of BRAF N581S, KRAS, PIK3CA E545K, microsatellite stable, and high tumor mutational burden (TMB) with 13 mutations per megabase. Given her performance status (PS) (ECOG PS 2), the side effects of regorafenib 120 to 160 mg, and TMB status, she started treatment with nivolumab 200 mg infusions every 2 weeks, plus oral regorafenib 80 mg once per day for 3 weeks in a 4-week cycle in October 2018. The combined treatment improved her PS (ECOG PS 1) and clinical symptoms (frequent urination and slow bowel movements) in as short as 2 courses. Significantly decreased CEA and CA19–9 levels were also seen. In April 2019, CT scans showed shrinkage of metastases in the lung, abdomen, and pelvis, with stable disease as the best response according to the Response Evaluation Criteria In Solid Tumors version 1.1. Meanwhile, disappearance of clinical symptoms and good PS of ECOG PS 0 were documented. With respect to toxicity, nivolumab plus regorafenib was generally tolerated with grade 3 hypothyroidism, grade 2 hand-foot syndrome, and grade 1 diarrhea.

**Figure 3:**
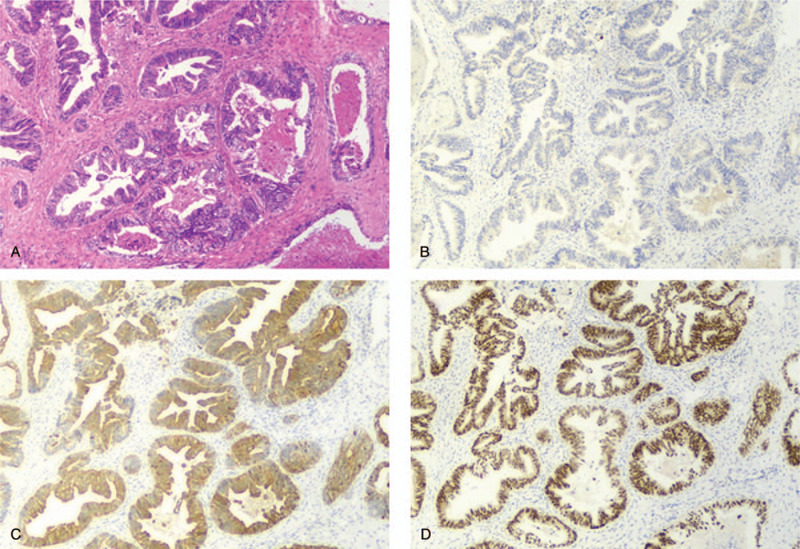
Pathologic findings of post-operative biopsies. Post-operative pathology showed a well-moderately differentiated adenocarcinoma (A, HE staining, x40), immunochemistry indicated (B) negative CK7, (C) positive CK20, and (D) positive CDX-2.

In September 2019, her condition deteriorated, with complaints of recurring frequent urination and slow bowel movements. CT scans displayed slightly enlarged lesions in the lung and pelvis. Subsequent palliative resection of pelvic, retroperitoneal, and peritoneal masses was conducted in October 2019. Postoperative biopsy (Fig. 3) showed a moderately differentiated adenocarcinoma with massive necrosis, and CK7 (–), CK20 (+), CDX-2 (+), mismatch-repair proficiency (pMMR). The patient continued to receive the combination of nivolumab and regorafenib. As of the time this article was written she was still alive and under treatment with nivolumab plus regorafenib with a progression-free survival (PFS) of 15 months and an OS of 51 months. The report was approved by the institutional review board of Beijing Huian Hospital of Integrated TCM and Western Medicine. Informed consent for publication was obtained from the patient.

## 3 Discussion

Advanced small bowel cancer carries a poor prognosis with a median survival time of around 1 year after diagnosis.^[4,6,7]^ Treatment options remain limited with surgery as the mainstay approach. Since small bowel cancer shares some characteristics with colon cancers, systemic therapy recommendations may be based on approaches to colon cancers. Frontline chemotherapy uses a combination of platinum salt and 5-FU (FOLFOX, CAPEOX, and folinic acid, 5-fluorouracil, oxaliplatin and irinotecan), which has a median OS of approximately 13.4 to 17.3 months and ensures a median time to progression or PFS of 5.9 to 9.4 months. ORR to frontline therapy range from 37.5% to 50%.^[10–13]^ There is a growing body of evidence that a favorable outcome can stem from the addition of anti- vascular endothelial growth factor receptor (VEGFR) or anti-endothelial growth factor receptor agent to chemotherapy without adding significant toxicity. CAPEOX plus bevacizumab yielded an ORR of 48.3%, a median PFS of 8.7 months and a median OS of 12.9 months in a prospective study.^[14]^ Anais L et al reported that 12% of SBA patients had at least 1 alteration of ERBB2, which was positively correlated with MSI status and APC mutations, and mutually exclusive with KRAS mutations.^[15]^ A case series indicated the potential role of anti-endothelial growth factor receptor therapy with cetuximab in SBAs, with the longest PFS of 10 months and the longest OS of 35 months.^[16]^

The current case was diagnosed in 2015, when the standard treatment had not been established yet. As CK20 (+)/CK7 (–) was observed, the first- and second-line therapy with oxaliplatin, irinotecan, and fluorouracil was given following the treatment of colon cancers. Some clinical benefits were obtained from these treatments with disease control of 12 to 27 weeks. Regorafenib monotherapy was administered as a subsequent treatment, since it was of greater benefit to survival than placebo (hazard ratio 0.77, *P* = .0052) in patients with metastatic colorectal cancer that progressed after all approved standard therapies.^[17]^ The PFS was 4 months for this patient, who, however suffered grade 3 toxicities with hand-foot syndromes and diarrhea which called for dose reduction. These findings suggest that regorafenib may provide an option for SBA patients, but tolerability and safety are a concern. Participation in clinical trials was recommended for this SBA patient, who received pemetrexed based on Lim et al report and participated in the clinical trial of DR5 antibody.^[18]^ Unfortunately, neither pemetrexed nor DR5 could halt progression of her disease and there was rapid progression in 1 to 2 courses.

The introduction of programmed death receptor-1/programmed death ligand-1 (PD-1/PD-L1) inhibitors into the treatment of gastrointestinal malignancies also shed light on the treatment of SBAs. Pembrolizumab and nivolumab are 2 PD-1/PD-L1 inhibitors which have been approved for treatment of dMMR/MSI-H colorectal cancer. In a cohort including 9 patients with mismatch repair-deficient non-colorectal cancers (including 2 SBA patients), the ORR and PFS rate at 20 weeks were 71% (5 of 7) and 67% (4 of 6), respectively, after pembrolizumab treatment.^[19]^ A phase 2 prospective study that evaluated the efficacy of pembrolizumab in SBA patients suggested that the disease control rate was 50% while only 8% of the patients achieved objective response.^[20]^ Nivolumab monotherapy showed an ORR of 31.1% in 74 patients with dMMR colorectal cancer, compared with 55% in patients treated with nivolumab plus ipilimumab.^[21]^ A possible synergistic effect with the combination of anti-angiogenesis and immunotherapy is also expected. VEGF-A/VEGFR blockage with VEGFR TKIs can enhance antitumor immunity via direct impact on immune cells such as dendritic cells and natural killer, modulate immunosuppression by downregulating regulatory T cells and myeloid-derived suppressor cells, inhibit tumor-Induced regulatory T-cell proliferation, and decrease PD-1 expression on T lymphocytes infiltrating the tumors.^[22–26]^ In clinic, a phase 1 REGONIVO study found that nivolumab plus regorafenib had an ORR of 40% and a PFS of 6.3 months in 50 patients with metastatic colorectal and gastric cancer progressing after standard therapies.^[27]^ Moreover, MSI-H/TMB-H, PD-L1 expression, and VEGF-A are frequently identified in SBAs, suggesting that this setting benefits from immunotherapeutic treatment.^[28–30]^ The current case was administered nivolumab plus oral regorafenib. The dose of regorafenib was reduced to 80 mg for fear of increased toxicity from this combination. She had stable disease with shrinkage of metastases and improved symptoms. The most serious adverse reaction was grade 3 hypothyroidism for this patient, which was relieved by levothyroxine. Other toxicities included grade 1 diarrhea and grade 2 hand-foot syndrome, which was consistent with other studies.

There is little data support for metastasectomy due to limited survival benefit. Resection of metastases is considered in case of emergency, such as bowel obstruction, bleeding or perforation.^[6]^. Metastasis to the ovary is less common than to the liver and peritoneal in advanced SBAs with an incidence of 1.6%.^[31]^ Ovary metastases are not sensitive to chemotherapy or radiotherapy, but surgical cytoreduction was reported to prolong survival in some cases with limited metastases.^[32,33]^ The current case enjoyed a 10-month local disease control from palliative resection of the ovary, suggesting that some SBA patients with limited metastases to the ovary may be candidates for metastasectomy.

As of the time this article was written, the patient had been able to keep the disease stable for 15 months. She remained active and was still receiving this combination. Consistent with the evidence currently available, the efficacy and tolerability of this case suggest the potential benefit of combining PD-1 inhibitor with VEGFR TKI in SBA patients.

## 4 Conclusion

In conclusion, our clinical experience suggests that dual blockage of VEGFR and PD-1/PD-L1 pathway with nivolumab and regorafenib may be effective against SBAs with prolonged survival. SBAs are rare and aggressive, hence the need for multidiscipline management involving a surgeon experienced in metastasectomy. Despite the clinical benefit from treatment for colorectal cancer, SBAs have distinct immunophenotype and molecular characterization.^[5,28]^ New targeted and immunotherapeutic treatment options are needed rather than translating regimens developed for other upper gastrointestinal malignancies alone.

## Acknowledgments

We would like thank our patient for her support.

## Author contributions

**Conceptualization:** Gairong Zhang, Li Lin, Ge Shen.

**Data curation:** Gairong Zhang, Hui Qiu, Tao Liu, Li Lian.

**Investigation:** Li Lin.

**Supervision:** Ge Shen.

**Visualization:** Gairong Zhang, Ge Shen.

**Writing – original draft:** Gairong Zhang, Dapeng Dong, Hui Qiu.

**Writing – review & editing:** Li Lin, Dapeng Dong, Hui Qiu, Tao Liu, Li Lian, Ge Shen.

## Abbreviations

Abbreviations: CA19–9 = carbohydrate antigen 19–9, CAPEOX = capecitabine and oxaliplatin, CEA = carcinoembryonic antigen, CT = computed tomography, FOLFOX = eucovorin calcium, 5-fluorouracil, and oxaliplatin, ORR = objective response rate, OS = overall survival, PD-1 = programmed death receptor-1, PD-L1 = programmed death ligand-1, PET-CT = positron emission computed tomography, PFS = progression-free survival, pMMR = mismatch-repair proficiency, PS = performance status, SBA = small bowel adenocarcinomas, TMB = tumor mutational burden, VEGFR = vascular endothelial growth factor receptor.
